# Observation on the Efficacy of Abdominal Massage in Treating Generalized Anxiety Disorder: A Randomized Controlled Trial Study Protocol

**DOI:** 10.7759/cureus.77030

**Published:** 2025-01-06

**Authors:** Haining Zhang, Huanan Li, Jingui Wang, An Bao

**Affiliations:** 1 Tuina Department, First Teaching Hospital of Tianjin University of Traditional Chinese Medicine, National Clinical Research Center for Chinese Medicine Acupuncture and Moxibustion, Tianjin, CHN

**Keywords:** abdominal massage, generalized anxiety disorder, gut-brain axis, gut microbiota, protocol, randomized controlled trial, tui na

## Abstract

Introduction and aim: Generalized anxiety disorder (GAD) is a prevalent mental health condition characterized by excessive, often uncontrollable anxiety, frequently accompanied by autonomic dysfunction symptoms. The gut-microbiota-brain axis is implicated in its pathogenesis. Traditional Chinese medicine therapies, including abdominal massage, are increasingly being considered for GAD treatment. This study is designed to evaluate the therapeutic potential of abdominal massage for GAD and its influence on patients' gut microbiota.

Materials and methods: A prospective, assessor-blinded, single-center randomized controlled trial involving 70 GAD patients, randomly allocated to either a combined abdominal massage and medication group or a medication-only group will be conducted. An additional 35 healthy individuals will serve as normal controls.

Results: The primary outcome measure will be the Hamilton Anxiety Rating Scale (HAMA) score pre- and post-treatment. Participants will undergo electroencephalogram (EEG) assessments and provide stool samples for gut microbiota evaluation. Differences between GAD patients and healthy controls will be compared, and changes in the two GAD groups pre- and post-treatment will be monitored. Subsequently, the correlation between EEG findings, gut microbiota, and clinical efficacy in GAD patients will be analyzed.

Conclusion: This study seeks to confirm the therapeutic benefits of abdominal massage for GAD and offer preliminary insights into its underlying mechanisms. Changes in these indicators before and after treatment in both the treatment and control groups will be examined to clarify the potential mechanisms by which abdominal massage may benefit GAD patients. Abdominal massage is hypothesized to alleviate clinical symptoms in GAD patients by modulating the gut-brain axis. If our hypothesis is validated, abdominal massage could emerge as a new alternative treatment for GAD and offer fresh insights into the mechanisms underlying massage therapy. The study's findings will be disseminated through peer-reviewed publications.

## Introduction

Generalized anxiety disorder (GAD) is a mental health condition primarily typified by excessive anxiety, frequently accompanied by symptoms of autonomic nervous system dysfunction [1]. The global lifetime prevalence of GAD is estimated at 3.7% [2]. In China, anxiety disorders' prevalence stands at 5.0%, with a lifetime prevalence reaching up to 7.6%, making it one of the most common mental health conditions [3]. GAD not only undermines the physical and mental health of the affected individuals but also leads to significant social and occupational dysfunction, posing a severe impact on societal and global health [4]. It is the most prevalent type of anxiety disorder [5], with symptoms primarily manifesting as tension, worry, fear, and autonomic dysfunction [6].

At present, the first-line treatment for GAD consists of pharmacotherapy, including benzodiazepine anxiolytics, receptor blockers, and selective serotonin reuptake inhibitors (SSRIs), among others [7]. However, the long-term use of these medications can result in dependence, addiction, an increased incidence of adverse drug reactions, and potential drug resistance, thereby diminishing treatment effectiveness [8]. Moreover, some patients may find the side effects of these medications intolerable and may opt to discontinue treatment. Consequently, there is a pressing need in clinical practice for a safe, effective, and easily applicable treatment method for GAD.

In recent years, the use of Traditional Chinese medicine (TCM) therapies in GAD management has been gaining traction. Particularly, TCM massage, and more specifically abdominal massage, is highly favored by patients due to its capacity to regulate internal organs, promote qi circulation, and balance yin and yang, all while being safe and comfortable [9,10]. Thus, understanding the mechanisms of abdominal massage and other unique TCM therapies has emerged as a significant area of academic interest and a crucial factor in advancing the application and development of TCM.

Objectives

This study aims to evaluate the therapeutic efficacy of abdominal massage in conjunction with escitalopram for the treatment of GAD. By correlating EEG metrics with anxiety assessment scales, a quantitative relationship between symptom scores and objective examination measures is aimed to be established. This will provide auxiliary diagnostic criteria and objective metrics for evaluating clinical efficacy in GAD.

Leveraging fecal 16S rDNA gene sequencing technology, this study will probe into the diversity, richness, and community structure of gut microbiota in GAD patients at a micro level. The regulatory effects of abdominal massage on gut microbiota will also be examined. This will further illuminate the gut-brain interaction mechanisms under the microbiota-gut-brain axis (MGBA) theory, demonstrating how abdominal massage contributes to the improvement of GAD.

## Materials and methods

Study design and setting

**Figure 1:**
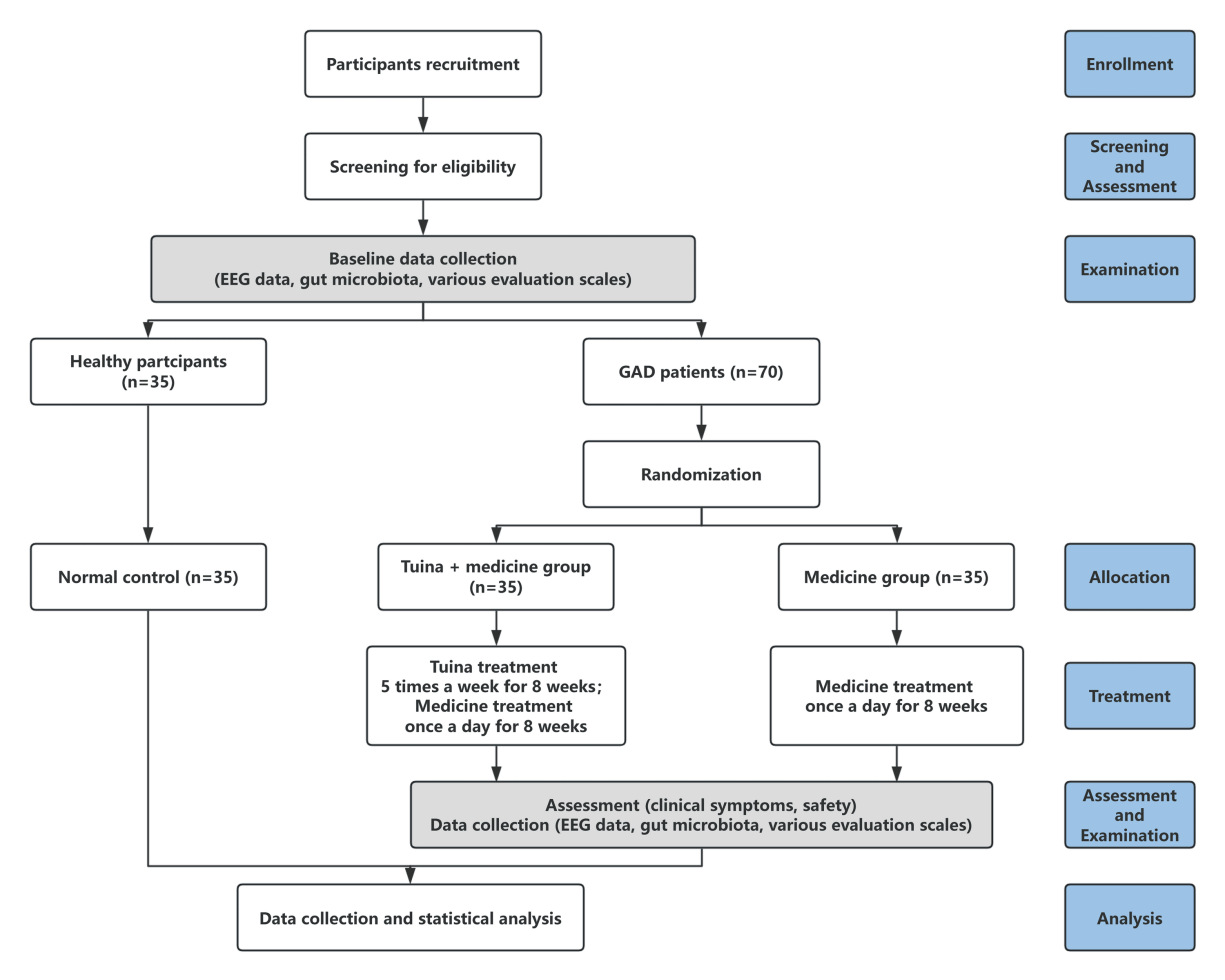
Flow diagram of the study participants enrollment and follow-up. EEG: electroencephalogram; GAD: generalized anxiety disorder

**Figure 2:**
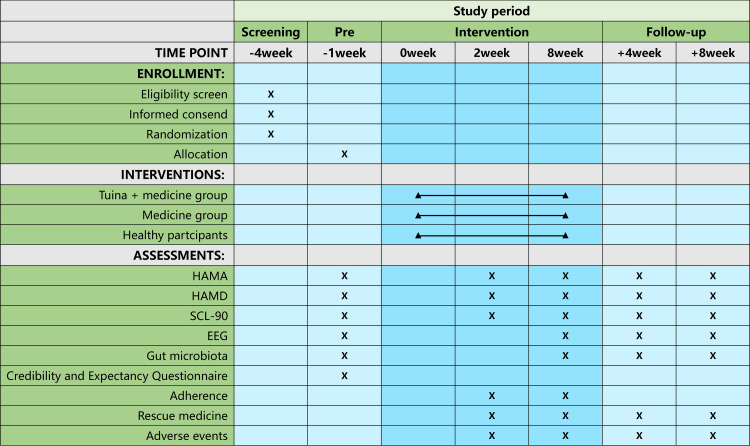
Schedule of enrollment, intervention, and assessments of this protocol. In the cells, "X" indicates that the examination was conducted at this time point; an empty cell signifies that the examination was not conducted at this time point. Cells connected by two triangles represent that treatment was administered during this period. HAMA: Hamilton Anxiety Scale; HAMD: Hamilton Depression Scale; SCL-90: Symptom Checklist 90; EEG: electroencephalogram

This study will be conducted as a parallel-designed, prospective, assessor-blinded, single-center randomized controlled trial. The timeline of this protocol study is from August 2024 to May 2025. This study will take place at the Department of Tui Na and the Psychosomatic Medicine Outpatient Department of the First Teaching Hospital of Tianjin University of Traditional Chinese Medicine, China. Seventy GAD patients will be randomly allocated to either the combined abdominal massage and medication group or the medication-only group. The study will involve various scale assessments, EEG examinations, and stool sample collections both pre- and post-intervention. Additionally, 35 healthy participants will be recruited to serve as normal controls and will undergo relevant tests and examinations upon enrollment. The trial flowchart is depicted in Figure 1, and the study timeline is outlined in Figure 2.

This randomized clinical trial is aligned with the principles of the Declaration of Helsinki. It has received approval from the Ethics Committee of the First Teaching Hospital of Tianjin University of Traditional Chinese Medicine (TYLL2024[Z]041) and has been registered on the International Traditional Medicine Clinical Trials Registry Platform (ITMCTR2024000140). All participants in this study will be voluntary participants and will sign informed consent forms prior to the commencement of the trial.

Participants

Diagnostic Criteria

The diagnostic criteria for Western medicine in this study are based on the International Classification of Diseases-11 (ICD-11) [11] and the Diagnostic and Statistical Manual of Mental Disorders-5 (DSM-5) [12]. The TCM diagnostic criteria draw on the 2008 Guidelines for the Diagnosis and Treatment of Common Diseases in Internal Medicine (TCM disease diagnosis section) [13] for depression (Appendix A).

Inclusion Criteria for GAD Patients

Participants must meet the previously mentioned Western and Traditional Chinese Medicine diagnostic criteria for GAD. They should be aged between 18 and 65 years, regardless of gender, with a HAMA score ranging from 14 to 29 prior to the trial, indicating mild to moderate GAD. Additionally, they must not have taken any other anti-anxiety or psychiatric medications in the past month and must voluntarily sign an informed consent form.

Exclusion Criteria for GAD Patients

Participants will be excluded if they have anxiety states induced by physical conditions such as hypertension, Sjögren's syndrome, hyperthyroidism, or coronary heart disease. Additionally, individuals currently taking medications that may cause anxiety or those experiencing withdrawal symptoms from long-term use of anti-anxiety medications will be excluded. Anxiety states linked to other mental disorders will also be grounds for exclusion. Patients with severe liver or kidney dysfunction who cannot adhere to the medication regimen, as well as those known to be allergic to any components used in this study, will not be eligible. Furthermore, pregnant or breastfeeding women will be excluded from participation.

Inclusion Criteria for Healthy Participants

Participants must be between 18 and 65 years old, regardless of gender, and should be right-handed. They must be in good health, without any physical discomfort in the past month. Individuals who have undergone central nervous system stimulation, such as repetitive transcranial magnetic stimulation (rTMS), transcranial direct current stimulation (tDCS), or peripheral nervous system stimulation, such as acupuncture or electroacupuncture, will be excluded. Additionally, candidates must have no history of illegal drug use or alcohol abuse. All participants must voluntarily sign the informed consent form and agree to take part in the survey.

Randomization and Assessor-Blinding

Patients with GAD will be randomly assigned to either the treatment group or the control group using computer-generated random numbers. The random sequences, identified with unique labels, will be sealed in opaque envelopes, which will only be opened upon the enrollment of a participant. The sequence generation and allocation will be carried out by independent personnel who are not involved in the trial. Healthy participants will be enrolled as normal controls, without undergoing randomization.

Blinded assessments will be performed, with patients receiving treatment in distinct settings. Efficacy will be evaluated by a third party who is not privy to the group assignments. During data aggregation, blinded statistical analysis will be conducted. Throughout the study, researchers, clinical operators, efficacy evaluators, and data statisticians will maintain separation to ensure the integrity of the blinding process.

Sample Size

Based on prior research, the Hamilton Anxiety Rating Scale (HAMA) score for the medication group was 18.61±3.17 [14]. The HAMA score for the treatment group is anticipated to decrease by 2.76 points. With a two-sided significance level (α) of 0.05 and a power of 90%, the required sample size was calculated using PASS 2021 software. This resulted in a needed sample size of N1=29 for the treatment group and N2=29 for the control group. Taking into account a potential dropout rate of 20%, a minimum of 35 subjects per group is needed, amounting to a total of at least 70 subjects. In addition, 35 healthy participants will be included in the study.

Intervention

Treatment Group GAD Patients

The medication regimen for this study will consist of oral escitalopram (Bailex) (Sichuan Kelun Pharmaceutical Co., Ltd., China; approval no.: National Drug Standard H20080788; specification: 10 mg × 10 tablets) [15]. Each participant will take 10 mg once daily at 09:00 AM for a duration of eight weeks.

In addition to the medication, participants in the treatment group will receive abdominal massages. The massage procedure will follow guidelines from the "Tui Na" textbook [16] edited by Fang Min and Wang Jingui (5th edition, "14th Five-Year Plan" for higher education in TCM). Participants will lie in a supine position while the practitioner performs rhythmic circular motions on the abdomen with the palm, 5 minutes clockwise and 5 minutes counterclockwise, at a frequency of 120 times/min. This is followed by finger massage on Zhongwan, Tianshu, Qihai, and Guanyuan points for 5 minutes at a frequency of 160 times/min. The total treatment time is approximately 15 minutes, conducted once daily, five days a week, for eight weeks.

To ensure the quality of the abdominal massage and the precise location of the acupoints, several measures will be implemented. Prior to the trial, Professor Wang Jingui's massage techniques, including force and frequency, will be calibrated using the YF-3 Hand Technique Measurement Instrument to create a standardized model. During the trial, experienced physicians with over 10 years of clinical practice will carry out the procedures. Additionally, all operators will receive training to ensure their techniques align with the model established by the YF-3 instrument.

After GAD patients are enrolled, they will initially receive two weeks of abdominal massage treatment by a Tui Na doctor, followed by training in the technique. After two weeks, the force applied by patients during self-massage will be measured to standardize the technique. For the remaining six weeks, patients will visit the clinic once a week for treatment, with the remaining sessions performed at home. Patients will receive a video tutorial on abdominal massage and are required to upload videos and photos of their self-treatment for review. If inconsistencies are found, the doctor will provide online guidance. If the technique is corrected after three attempts, the patient will remain in the study; otherwise, they will be considered a dropout.

**Figure 3:**
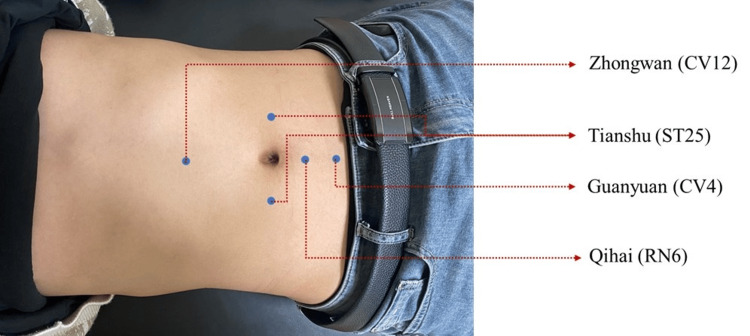
Acupoint location diagram.

The acupoints utilized in this study are defined according to the 2021 national standard (GB/T12346-2021) titled "Names and Locations of Acupoints."[17] These acupoints include Zhongwan (CV12), which is situated in the upper abdomen, 4 cun above the navel along the anterior midline; Tianshu (ST25), located at the level of the navel, 2 cun lateral to the anterior midline; Qihai (RN6), found in the lower abdomen, 1.5 cun below the navel on the anterior midline; and Guanyuan (CV4), positioned 3 cun below the navel on the anterior midline. A map illustrating the locations of these acupoints is provided in Figure 3.

Control Group GAD Patients

Medication includes oral escitalopram (Bailex) (Sichuan Kelun Pharmaceutical Co., Ltd., China; approval no.: National Drug Standard H20080788; specification: 10 mg × 10 tablets), 10 mg once daily at 09:00 AM for eight weeks.

Healthy Participants

Healthy participants will not receive any treatment. They will be screened based on their medical history and HAMA scores. General information and HAMA scores will be recorded. Upon enrollment, EEG and stool samples will be collected from healthy participants.

Statistical analysis

This study will conduct statistical analysis based on the principle of intention-to-treat (ITT), including all patients who entered randomization and were not excluded in the statistical analysis. The efficacy endpoints will be analyzed using the full analysis set (FAS), supplemented by the per-protocol set (PP). Safety endpoints will be analyzed using the safety set (SS).

IBM SPSS Statistics for Windows, Version 26 (Released 2019; IBM Corp., Armonk, New York, United States) will be used for statistical processing. Measurement data will first be tested for normal distribution and homogeneity of variance. Data that meet the normal distribution and homogeneity of variance will be statistically described using the mean ± standard deviation (x̄±s), and data that do not meet the normal distribution and homogeneity of variance will be statistically described using the median (M) and the 25th and 75th percentiles (P_25_, P_75_).

For the comparison of measurement data between two groups, a paired two-sample t-test will be used for data that meet the normal distribution, and a rank-sum test will be used for data that do not meet the normal distribution. For the comparison of data at different time points after treatment with pre-treatment data, a paired t-test will be used for data that meet the normal distribution, and a rank-sum test will be used for data that do not meet the normal distribution. For the comparison of measurement data among multiple groups, a one-way ANOVA will be used for data that meet the normal distribution, and a rank-sum test will be used for data that do not meet the normal distribution.

For the comprehensive comparison of measurement data at different time points between two groups, a repeated measures ANOVA will be used if the data meet the sphericity assumption and a Friedman test will be used if the data do not meet the normal distribution. Count data will be statistically described using case number and composition ratio, and the comparison between groups will be performed using the χ² test. Rank data will be analyzed using the two-sample non-parametric rank-sum test. The significance level for all tests will be α=0.05.

## Results

Primary outcome

The primary outcomes of this study will be determined by the HAMA [18] scores and electroencephalogram (EEG) results. The HAMA scale consists of 14 items, which will be evaluated by professional psychiatrists before and after the treatment. The total HAMA score can range from 0 to 56, with anxiety levels classified as follows: possible anxiety for scores between 7 and 14, definite anxiety for scores between 14 and 21, moderate anxiety for scores between 21 and 29, and severe anxiety for scores greater than 29.

For the EEG, participants will be seated in a quiet environment, with their eyes closed, while maintaining an alert state. Scalp electrodes will be placed according to the international 10-20 system, using 17 disc electrodes with earlobes serving as reference electrodes. The EEG will record both bipolar and reference leads, with standard evoked tests (including hyperventilation, rhythmic photic stimulation, and eye-opening/closing tests) conducted during monitoring. The time constant will be set at 0.3 ms, the high-frequency filter will be set at 70 Hz, the standard voltage will be set at 10 mV/mm, and the recording time will be between 15 and 20 minutes.

Other outcome measures

Other outcome measures in this study include symptom signs, scores on the Hamilton Depression Rating Scale (HAMD), scores on the Symptom Checklist-90 (SCL-90), and results from monitoring the gut microbiota.

Hamilton Depression Rating Scale

This scale is used to assess the severity of depression in patients and consists of 17 items. Depression severity is classified based on HAMD scores as follows: a score of less than 7 suggests no depression; a score between 7 and 16 suggests possible depression; a score of 17 or greater indicates definite depression.

Symptom Checklist-90

This scale is used to evaluate patients' self-perceived symptoms during treatment. Each question is rated on a scale from 1 (none) to 5 (severe), with higher scores indicating more severe adverse symptoms.

Gut Microbiota Monitoring

Stool samples will be collected from randomly selected patients in the treatment and control groups before and after eight weeks of treatment. Samples will be collected in the morning, on an empty stomach, using a sterile spoon to obtain 3-5 g of fresh stool. These samples will be immediately placed in standardized sterile containers and stored at -80°C for subsequent analysis [19].

Safety assessments

All participants will undergo physical examinations and vital signs testing. Any adverse events will be documented in detail, and participants will receive appropriate treatment immediately. Most adverse events related to abdominal massage are mild, such as abdominal soreness, which typically resolves with rest. Severe adverse events will be promptly reported to the Ethics Committee of the First Teaching Hospital of Tianjin University of Traditional Chinese Medicine. Participants will decide whether to continue in the trial if adverse events occur.

Quality control, data management, and monitoring

Before the commencement of the trial, a clinical research manual will be prepared, and all researchers will undergo professional training to ensure their familiarity with the clinical research protocol and consistency in efficacy evaluations. Standard procedures for Tui Na operations will be established to ensure accuracy, and Tui Na practitioners will be periodically supervised.

All original patient data will be retained in case report forms, which will be stored in a secure, restricted environment. A researcher, who will not be privy to group assignments, will enter the data into a pre-designed, password-protected electronic database. Access to this database will be granted only to designated members of the research team.

To ensure privacy and confidentiality, all research documents will be stored in a locked cabinet located in a secured office in Chengdu. The Ethics Committee of the First Teaching Hospital of Tianjin University of Traditional Chinese Medicine may inspect research records and monitor the trial process to ensure adherence to ethical standards.

## Discussion

This study is a randomized controlled trial designed to investigate the therapeutic effects of abdominal massage (Tui Na) on GAD and explore the relationship between clinical efficacy and the gut MGBA.

The pathogenesis of GAD is complex and not fully understood, involving factors such as genetic predisposition, neurotransmitter changes, neuroendocrine dysfunction, immune system abnormalities, and disruptions in gut microbiota. With the advancement of gut microbiota research, the "gut microbiota-gut-brain axis" has emerged as a promising field in the study of mental and emotional disorders. This axis refers to the close connection between gut function and brain emotional cognition through neurotransmitter, endocrine, immune, and metabolic pathways, playing a crucial role in regulating behavior and emotions [20,21]. The brain can also regulate the structure and composition of gut microbiota to maintain intestinal microecological balance. The close relationship between gut microbiota and mental-emotional disorders has been increasingly confirmed by scientific experiments, which is significant for elucidating the pathogenesis of GAD and seeking new treatment methods.

In TCM, GAD can be categorized under the scope of "Yu Zheng" (depressive disorder), often caused by the blockage of Qi movement [22]. TCM recognizes the close relationship between the spleen, stomach, and emotions. The spleen and stomach are the pivot of Qi movement, and abdominal massage can directly regulate spleen and stomach functions, thereby adjusting the Qi movement, nourishing the heart, and calming the mind. The acupuncture points Zhongwan, Qihai, Guanyuan, and Tianshu, located on the abdomen, are crucial for strengthening the spleen and stomach and regulating Qi movement. Modern clinical research has also demonstrated that abdominal massage for GAD is comparable to Western medication in efficacy, with no adverse effects, and has significant advantages in alleviating accompanying symptoms such as bloating and loose stools [23,24]. Therefore, abdominal massage shows promise as an effective adjunctive therapy for treating GAD.

This study aims to design a high-quality randomized controlled trial to observe changes in brain electrical activity and clinical efficacy indicators before and after the combined treatment of abdominal massage and escitalopram in GAD patients. EEG recordings will be used to analyze the spontaneous and rhythmic electrical activity of brain cell groups, focusing on brain discharge indices and changes in alpha rhythms. Additionally, fecal 16S rDNA gene sequencing technology will be employed to examine the diversity, richness, and community structure of gut microbiota in GAD patients, as well as the regulatory effects of abdominal massage on these microbiota. This will help further elucidate the brain-gut interaction mechanism through which abdominal massage improves GAD based on the gut-brain axis theory.

However, this study has certain limitations in its design. Firstly, due to the nature of the intervention, it is impossible to blind the patients and the massage therapists. To minimize performance and detection biases, allocation concealment will be implemented, and outcome measurements will be conducted by assessors who are unaware of group assignments and interventions. Secondly, a fixed massage treatment protocol will be used for all participants in the trial without differentiation, which may not fully demonstrate the efficacy of the massage. Additionally, the sample size is relatively small. These potential limitations might affect the results and necessitate future studies.

## Conclusions

This is the first randomized controlled trial to incorporate abdominal massage into the gut-brain axis framework for GAD patients. EEG and gut microbiota are indicators of gut-brain axis function. Differences in these indicators between GAD patients and healthy individuals will be identified to determine the characteristics of gut-brain interactions in GAD patients. Furthermore, changes in these indicators before and after treatment in the treatment group and the control group will be studied to elucidate the potential mechanisms of abdominal massage in treating GAD patients. The main advantage of this study is the collection and evaluation of multifaceted data. Abdominal massage is hypothesized to improve clinical symptoms in GAD patients by regulating the gut-brain axis. If this hypothesis is confirmed, abdominal massage will not only become a new alternative method for treating GAD but also provide new insights into the mechanisms of massage therapy.

## Appendices

Appendix A 

**Table 1:** Diagnostic criteria for Western and traditional Chinese medicine.

Diagnostic classification	Diagnostic entries
Western medicine diagnostic criteria	Excessive worry and anxiety about events and activities in work or life for at least six months.
	Difficulty controlling this anxiety or worry.
	At least three of the following symptoms are present: feeling restless, agitated, or continuously tense; easily fatigued; difficulty concentrating; easily irritated; muscle tension; sleep disturbance.
	This anxiety or worry, or physical symptoms, cause clinically significant distress or lead to social, occupational, or other important functional impairments.
	This disorder cannot be attributed to the physiological effects of a substance (such as a drug or medication) or other physical disease, such as hyperthyroidism, etc.
	This disorder cannot be well explained by other mental disorders (such as the negative evaluation in social anxiety disorder, contamination obsessions in obsessive-compulsive disorder, separation from attachment objects in separation anxiety disorder, etc.).
Traditional Chinese medicine diagnostic criteria	The main clinical symptoms are melancholy, restlessness, chest and rib distension and pain, or easy crying and anger, emotional variability, or feeling of obstruction in the throat.
	There is often a history of emotional injuries such as worry, anxiety, sadness, fear, and anger, and the recurrence of the disease is often related to emotional changes caused by various factors.
	Examinations of various systems and physical and chemical examinations are normal, which can exclude organic diseases.
